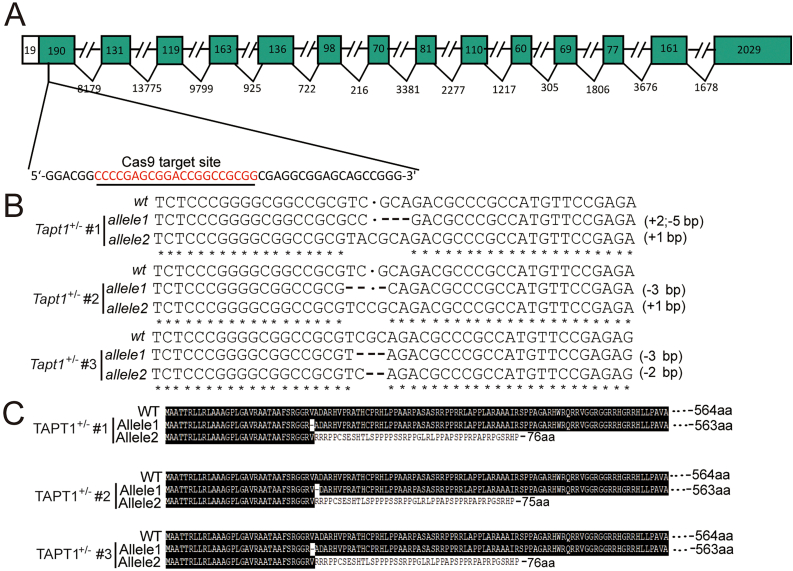# Transmembrane anterior posterior transformation 1 regulates BMP signaling and modulates the protein stability of SMAD1/5

**DOI:** 10.1016/j.jbc.2022.102684

**Published:** 2022-11-09

**Authors:** Bo Wang, Qian Zhao, Xiaoxia Gong, Caixia Wang, Yan Bai, Hongying Wang, Jianfeng Zhou, Xiaozhi Rong

**Affiliations:** 1 Key Laboratory of Marine Drugs (Ocean University of China), Chinese Ministry of Education, and School of Medicine and Pharmacy, Ocean University of China, Qingdao, China; 2 Laboratory for Marine Drugs and Bioproducts, Pilot National Laboratory for Marine Science and Technology (Qingdao), Qingdao, China; 3 Hubei Provincial Key Laboratory for Protection and Application of Special Plants in Wuling Area of China, Key Laboratory of State Ethnic Affairs Commission for Biological Technology, College of Life Sciences, South-Central Minzu University, Wuhan, China

**Keywords:** TAPT1, bone morphogenetic protein, SMAD transcription factor, SMURF1, C2C12, C3H/10T1/2, ossification, BMP, bone morphogenetic protein, ALP, alkaline phosphatase, co-IP, coimmunoprecipitation, DV, dorsoventral, FBS, fetal bovine serum, GOF, gain-of-function, hpf, hours post fertilization, SMURF1, SMAD ubiquitin regulatory factor 1, TAPT1, Transmembrane anterior posterior transformation 1

## Abstract

The bone morphogenetic protein (BMP) signaling pathway plays pivotal roles in various biological processes during embryogenesis and adult homeostasis. Transmembrane anterior posterior transformation 1 (TAPT1) is an evolutionarily conserved protein involved in murine axial skeletal patterning. Genetic defects in TAPT1 result in complex lethal osteochondrodysplasia. However, the specific cellular activity of TAPT1 is not clear. Herein, we report that TAPT1 inhibits BMP signaling and destabilizes the SMAD1/5 protein by facilitating its interaction with SMURF1 E3 ubiquitin ligase, which leads to SMAD1/5 proteasomal degradation. In addition, we found that the activation of BMP signaling facilitates the redistribution of TAPT1 and promotes its association with SMAD1. TAPT1-deficient murine C2C12 myoblasts or C3H/10T1/2 mesenchymal stem cells exhibit elevated SMAD1/5/9 protein levels, which amplifies BMP activation, in turn leading to a boost in the transdifferentiation or differentiation processing of these distinct TAPT1-deficient cell lines changing into mature osteoblasts. Furthermore, the enhancing effect of TAPT1 deficiency on osteogenic differentiation of C3H/10T1/2 cells was observed in an *in vivo* ectopic bone formation model. Importantly, a subset of TAPT1 mutations identified in humans with lethal skeletal dysplasia exhibited gain-of-function activity on SMAD1 protein levels. Thus, this finding elucidates the role of TAPT1 in the regulation of SMAD1/5 protein stability for controlling BMP signaling.

Bone morphogenetic proteins (BMPs) are multifunctional growth factors, which control a wide range of biological processes in both invertebrate and vertebrate during embryonic development and adult homeostasis (1). In vertebrate embryos, cell fates are patterned along the dorsoventral (DV) axis, with BMP signals forming a ventral-to-dorsal gradient from high to low levels to control DV patterning (2, 3, 4, 5). In addition, BMP signaling plays a key role in tissue homeostasis by regulating the cell proliferation, differentiation, cell-fate determination, and cell death (1).

Upon BMP ligand-induced assembly of the receptor complex, the BMP type II receptor acts as a constitutively active kinase to phosphorylate and activate the type I receptor. Subsequently, activated type I receptor kinases phosphorylate the receptor-regulated intracellular effector SMADs (R-SMADs) (SMAD1/5), at two C-terminal serines (6). The phosphorylated and activated R-SMADs associate with SMAD4 and then translocate into the nucleus. Within the nucleus, SMAD complexes interact with various transcription factors to regulate target gene transcription (7, 8, 9).

The duration of SMAD1 activation is a key in the output of the BMP signaling pathway. The linker region of SMAD1 contains conserved MAPK and GSK3 recognition sites which were sequentially phosphorylated by MAPK and GSK3 to control BMP signal intensity (10, 11). After multiple phosphorylation, SMAD1 is ultimately recognized by E3 ubiquitin ligases, such as SMAD ubiquitin regulatory factor 1 (SMURF1), for ubiquitination and proteasomal degradation (11).

Transmembrane anterior posterior transformation 1 (TAPT1) was named so because *Tapt1*-mutant mice exhibit posterior-to-anterior transformations of the vertebral column midsection (12, 13). Although the mutant phenotype is specific, the *Tapt1* expression pattern is ubiquitous in both the whole E7–E17 embryos and adult tissue (12). In addition, genetic mutations in *TAPT1* cause complex lethal skeletal dysplasias and ciliopathies with severe hypomineralization of the entire skeleton as well as intrauterine fractures (14). However, the cellular functions and molecular mechanisms of TAPT1 are poorly understood.

Considering that BMP signaling plays a vital role in normal skeletal development, we hypothesized that TAPT1 may function as a regulator of the BMP signaling pathway. In the current study, we tested this hypothesis and investigated the role of TAPT1 in the BMP signaling pathway both *in vitro* and *in vivo*. We found that TAPT1 binds to SMAD1/5, reduces protein levels, and inhibits BMP signaling in the presence of BMP signals. In addition, BMP treatment promoted the association between SMAD1 and TAPT1. Mechanistic studies demonstrated that TAPT1 binds to SMAD1/5 as well as to SMURF1 and promotes their association, which in turn facilitates SMAD1/5 proteasomal degradation. This process occurs both within the cytoplasm and within the nucleus. TAPT1 deficiency increases the protein levels of SMAD1/5/9, thus promoting the ossification of C2C12 and C3H/10T1/2 cells under BMP treatment. Our results also suggested that two lethal osteochondrodysplasia-associated mutations of *TAPT1* likely function as gain-of-function (GOF) variants to promote SMAD1 proteasomal degradation. The current findings highlight the importance of TAPT1 as a BMP inhibitor, which acts *via* binding to and promoting SMAD1/5 proteasomal degradation.

## Results

### Forced expression of TAPT1/Tapt1 in zebrafish dorsalizes embryos and inhibits Bmp signaling

**Figure 1:**
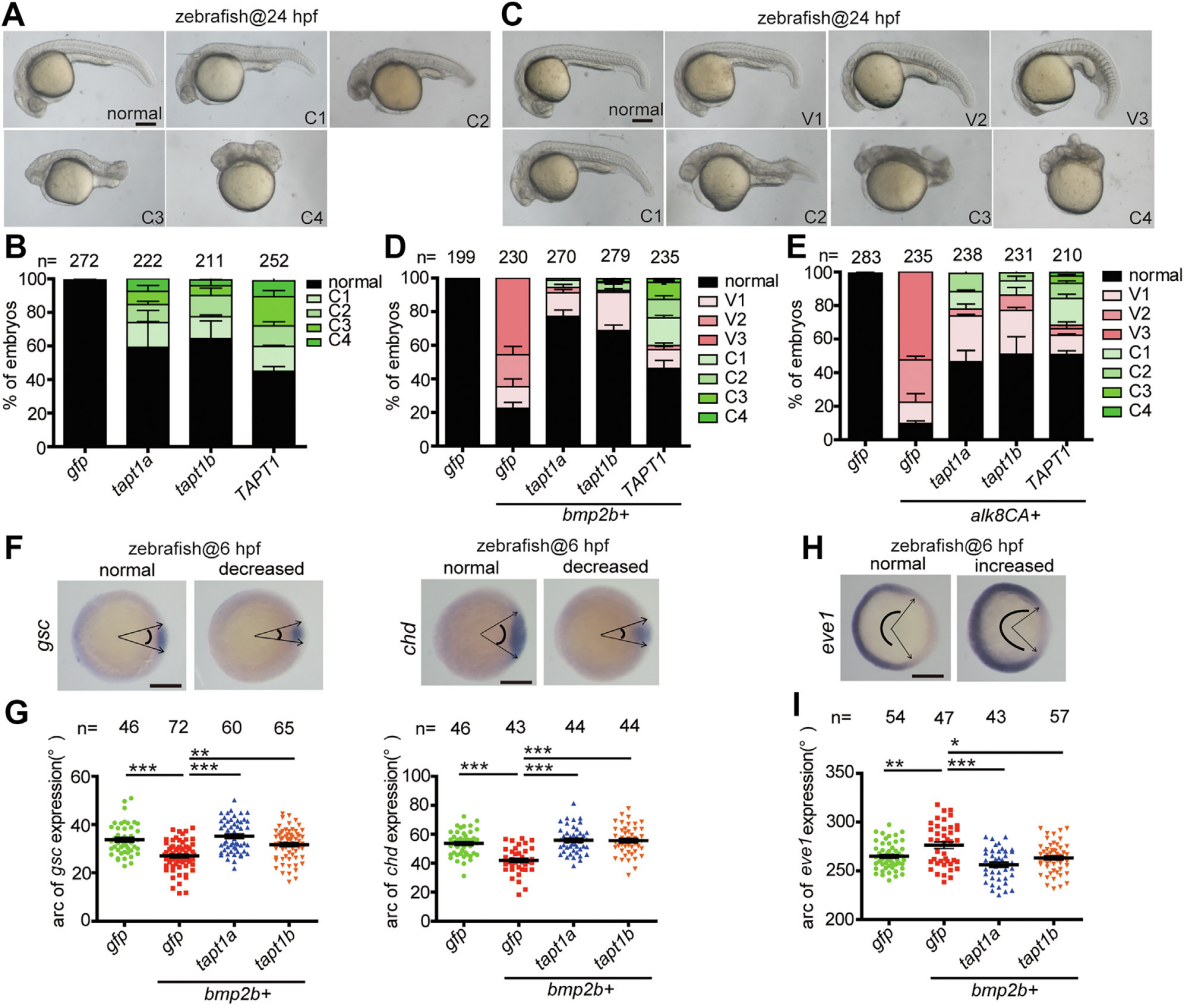
**Overexpression of TAPT1/Tapt1 in zebrafish caused dorsalized phenotypes by inhibiting Bmp signals.***A*, phenotypic classification of embryos at 24 hpf caused by the forced expression of mRNA for human *TAPT1* or zebrafish *tapt1a/tapt1b*. *B*, quantitative result shown in (*A*). The embryos were injected with 800 pg of *tapt1a* mRNA or 600 pg of *TAPT1* or *tapt1b* mRNA. *C*–*E*, the antagonizing effect of *TAPT1*, *tapt1a*, or *tapt1b* mRNA on Bmp2b- and Alk8CA-induced Bmp signaling actions *in vivo*. Representative images of embryos injected with 610 pg of *gfp* mRNA, 10 pg of *bmp2b* or *alk8CA* mRNA, and mRNA of each plus 600 pg of *TAPT1* or *tapt1a/1b* mRNA at 24 hpf are shown in (*C*). Quantitative results are shown in *D* and *E*. *F* and *H*, expression patterns of dorsoventral markers in *bmp2b* mRNA- or *bmp2b* mRNA plus *tapt1a or tapt1b* mRNA-injected embryos at 6 hpf. The edges of specific markers are indicated by *arrows*. *Top* views with dorsal to the *right*. *G* and *I*, quantification of the arc of marker expression shown in (*F* and *H*). The total number of embryos of each group are given at the *top*. Similar results were obtained from three experiments. Data are from three independent experiments with individual data points shown. Values are means ± S.D. ∗*p* < 0.05; ∗∗*p* < 0.01; ∗∗∗*p* < 0.001. Unpaired *t* test, two-tailed. The scale bars represent 200 μm. Bmp, bone morphogenetic protein; hpf, hours post fertilization; TAPT1, Transmembrane anterior posterior transformation 1.

**Supplementary Figure 1:**
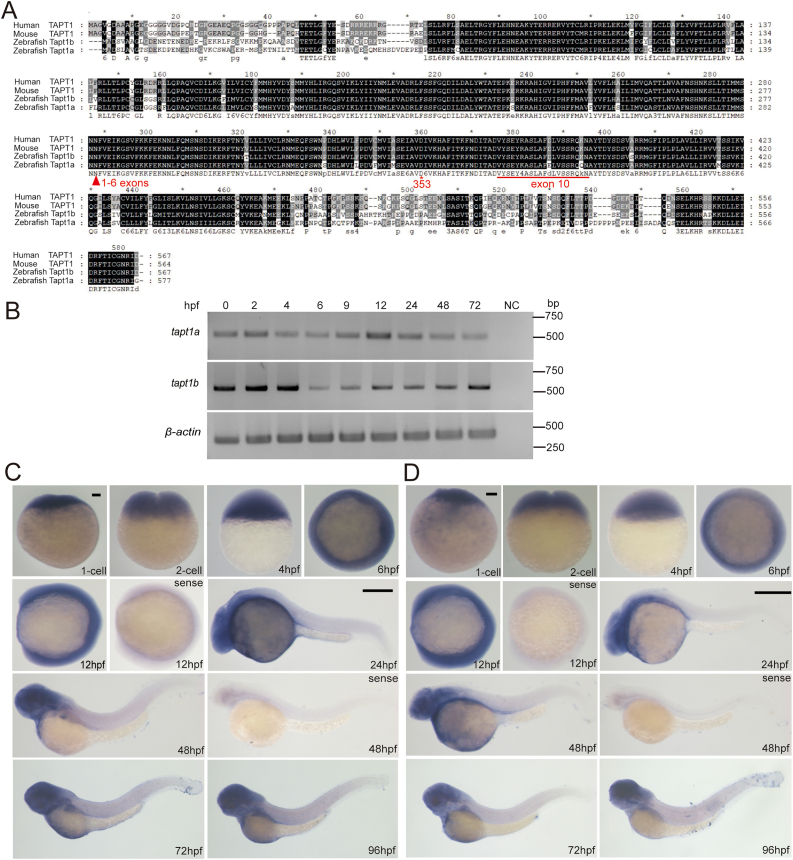


Numerous studies have shown that the zebrafish embryo is an excellent *in vivo* model for investigating the function of Bmp signaling, as Bmp proteins act as morphogens to pattern the DV axis (2, 15). Excessive Bmp signals ventralize zebrafish embryos, whereas insufficient Bmp signals dorsalize embryos. The human and mouse genomes contain one *TAPT1/Tapt1* gene, while the zebrafish genome contains two *tapt1* genes, *tapt1a* and *tapt1b*. TAPT1 is highly conserved between zebrafish and humans (Fig. S1*A*). We performed RT-PCR and whole-mount *in situ* hybridization analysis to examine the spatiotemporal expression pattern of *tapt1a* and *tapt1b* in zebrafish during embryogenesis. Both were maternally deposited and ubiquitously expressed before 24 h post fertilization (hpf) (Fig. S1, *B*–*D*). To investigate the effects of Tapt1a and Tapt1b in zebrafish embryos, we forced the expression of zebrafish *tapt1a* and *tapt1b* mRNAs into 1-2 cell stage zebrafish embryos and then raised them to 24 hpf. Overexpression of zebrafish Tapt1a and Tapt1b resulted in dorsalized phenotypes at 24 hpf along with different categories (C1-C4), including a shortened tail or a truncated body plan with loss of tail (Fig. 1, *A* and *B*) (16). Human *TAPT1* had a comparable dorsalizing effect (Fig. 1, *A* and *B*). Previous studies have described that genetic mutations in human *TAPT1* or mouse *Tapt1* lead to abnormal bone development (12, 13, 14). These findings prompted us to investigate whether TAPT1 is involved in BMP signaling. Ectopically enhanced BMP signaling in zebrafish embryos results in variable degrees of ventralization (17, 18). However, forced expression of human *TAPT1* or zebrafish *tapt1a*/*tapt1b* mRNAs dorsalized zebrafish embryos. Therefore, we examined whether TAPT1 or Tapt1a/Tapt1b can antagonize the action of Bmp signaling during formation of the DV axis. In agreement with previous results, injection of *bmp2b* or *alk8CA* (Q204D, the constitutively active mutant of the type I receptor Alk8) resulted in ventralized embryos at 24 hpf with different degrees (V1-V3) (Fig. 1, *C*–*E*) (18, 19). Coinjection of human *TAPT1* mRNA with *bmp2b* or *alk8CA* mRNA antagonized the Bmp2b- and Alk8CA-induced ventralizing effects in zebrafish embryos (Fig. 1, *C*–*E*). Consistently, zebrafish Tapt1a and Tapt1b had a similar effect (Fig. 1, *C*–*E*). To confirm the antagonizing effect of TAPT1 on Bmp signaling–induced ventralization, we performed whole-mount *in situ* hybridization with dorsoventral markers. Injection of *bmp2b* mRNA into zebrafish embryos reduced the expression areas of dorsal genes *chd* and *gsc* and increased the expression domains of ventral gene *eve1* at the shield stage (Fig. 1, *F*–*I*). Coinjection of *tapt1a* or *tapt1b* mRNA with *bmp2b* mRNA reversed the Bmp2b-induced reduction in *chd* and *gs*c, as well as the expansion of *eve1* at the shield stage (Fig. 1, *F*–*I*). Taken together, these results suggested that overexpression of TAPT1/Tapt1 dorsalizes zebrafish embryos and antagonizes BMP signaling during the formation of DV patterning.

### TAPT1 inhibits BMP signaling by reducing SMAD1/5 protein levels

**Figure 2:**
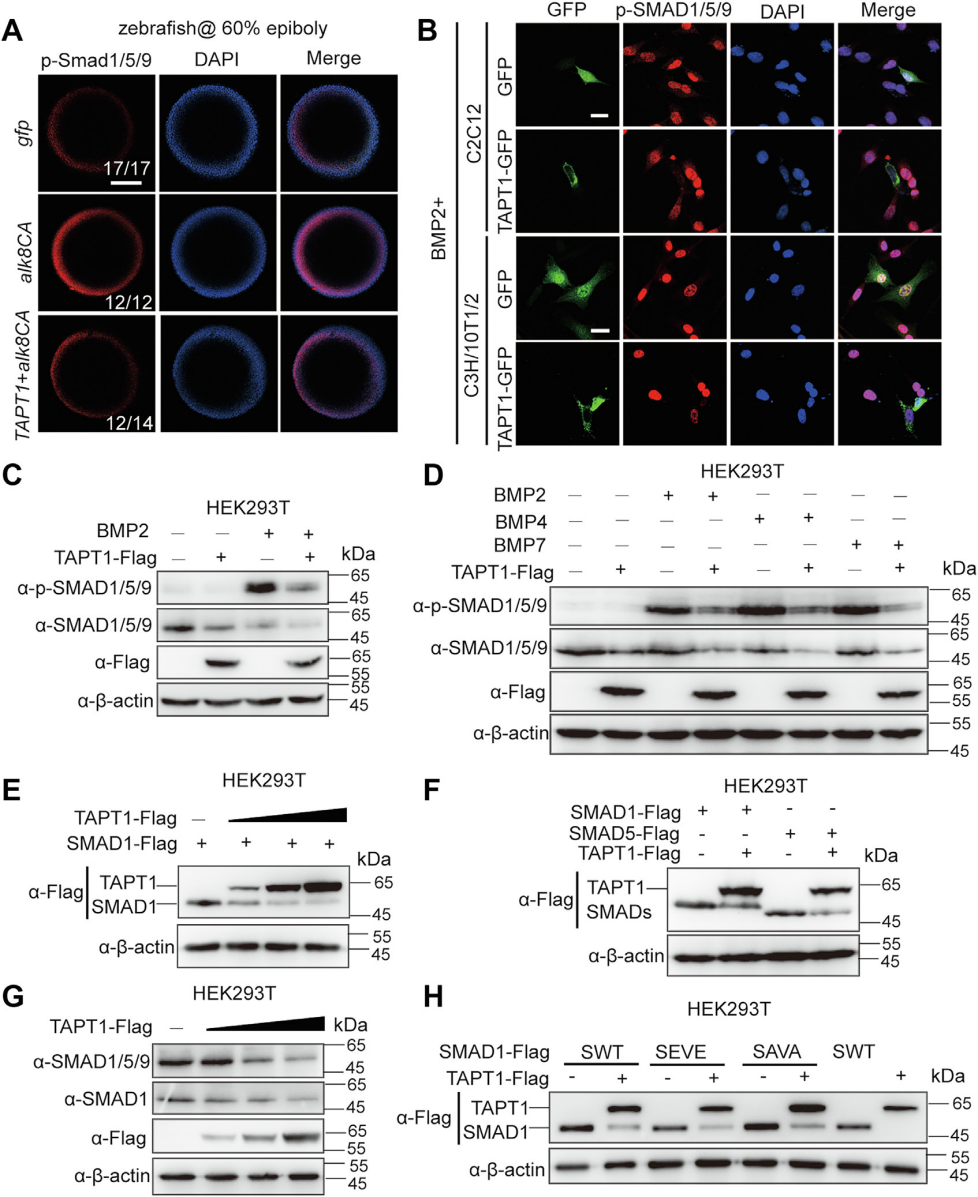
**TAPT1 inhibits BMP signaling by reducing SMAD1/5 protein levels.***A*, immunostaining images for p-Smad1/5/9 in indicated zebrafish embryos. Embryos at 1-2 cell stage were injected with 610 pg of *gfp* mRNA, 10 pg of *alk8CA* mRNA, or 10 pg of *alk8CA* mRNA plus 600 pg of *TAPT1* mRNA and then raised to 60% epiboly stage for immunostaining with an anti-p-SMAD1/5/9 antibody (*red*). Nuclei were counterstained with DAPI (*blue*). *Top* views with dorsal to the *right*. The total number of embryos of each group are given at the *right bottom corner*. The scale bar represents 200 μm. *B*, the nuclear accumulation of p-SMAD1/5/9 in GFP-overexpressing or GFP-TAPT1–overexpressing C2C12 or C3H/10T1/2 cells under BMP2 treatment. GFP-tagged TAPT1 or GFP expression vector was transfected into C2C12 or C3H/10T1/2 cells. After 24 h, cells were starved with serum-free medium for 1 h, subsequently stimulated with 100 ng/ml BMP2 protein for another 1 h, and then the immunostaining was performed with the antibody to p-SMAD1/5/9 (*red*). Nuclei were labeled with DAPI (*blue*). The scale bar represents 20 μm. *C*, endogenous protein levels of p-SMAD1/5/9 and total SMAD1/5/9 in control or TAPT1-overexpressing HEK293T cells with or without BMP2 treatment. HEK293T cells were transfected with Flag-tagged TAPT1 or an empty vector. After 24 h, cells were starved with serum-free medium for 1 h and subsequently stimulated with or without 100 ng/ml BMP2 protein for another 1 h. Similar results were obtained from three experiments. *D*, endogenous protein levels of p-SMAD1/5/9 and total SMAD1/5/9 in TAPT1-overexpressing HEK293T cells with BMP2, BMP4, or BMP7 treatment. After transfection with Flag-tagged TAPT1, cells were starved for 1 h and then treated with BMP2, BMP4, or BMP7 protein for another 1 h. *E*, exogenous SMAD1 protein levels in control or different doses of TAPT1-overexpressing HEK293T cells. *F*, the exogenous protein levels of SMAD1 or SMAD5 in control or TAPT1-overexpressing HEK293T cells. SMADs were transfected into HEK293T cells with Flag-TAPT1 or an empty vector. *G*, endogenous SMAD1 and SMAD1/5/9 protein levels in control or different doses of TAPT1-overexpressing HEK293T cells. *H*, WT SMAD1, SEVE mutant, and SAVA mutant protein levels in control or TAPT1-overexpressing HEK293T cells. SMAD1 constructs encoding WT SMAD1 (SWT), a BMP-independent phospho-mimetic–activated SMAD1 (SEVE), and nonphospho-mimetic–inactivated SMAD1 (SAVA) were transfected into HEK293T cells with Flag-tagged TAPT1 or an empty vector. BMP, bone morphogenetic protein; TAPT1, Transmembrane anterior posterior transformation 1.

In response to the activation of BMP signaling, Smad1/5/9 is phosphorylated by the BMP type I receptor. In zebrafish, p-Smad1/5/9 forms a high-to-low activity gradient from the ventral side to the dorsal side to control DV patterning (20, 21, 22, 23). To further test whether TAPT1 inhibits BMP signaling, we coinjected *TAPT1* and *alk8CA* into 1-2 cell stage embryos and then monitored p-Smad1/5/9 levels along the DV axis of embryos at 60% epiboly. Injection of *alk8CA* mRNA significantly increased the ventral to dorsal expression levels of p-Smad1/5/9 (Fig. 2*A*). Compared with *alk8CA* mRNA-injected embryos, TAPT1- and Alk8CA-coexpressed embryos at 60% epiboly showed relatively weak p-Smad1/5/9 signals (Fig. 2*A*). This result indicated that overexpression of TAPT1 reduced Bmp signaling–induced p-Smad1/5/9 levels in zebrafish embryos.

To further confirm this result, C2C12 cells were transfected with GFP alone or GFP-tagged TAPT1, followed by BMP2 treatment. Next, we performed immunostaining analysis with an antibody against p-SMAD1/5/9 to examine p-SMAD1/5/9 levels in TAPT1-overxpressing C2C12 cells. Compared with BMP2-induced strong nuclear p-SMAD1/5/9 signals in GFP-expressing cells, weak or barely detectable p-SMAD1/5/9 signals were observed in GFP-tagged TAPT1-expressing C2C12 cells after treatment with BMP2 (Fig. 2*B*, upper panel). Similar result was observed in C3H/10T1/2 cells (Fig. 2*B*, below panel). Moreover, we found that overexpression of TAPT1 decreased the protein levels not only of phosphorylated SMAD1/5/9 induced by BMP2 but also of total SMAD1/5/9 without BMP2 treatment in HEK293T cells (Fig. 2*C*). Similarly, overexpression of TAPT1 also decreased the protein levels of phosphorylated SMAD1/5/9 induced by BMP4 and BMP7, two osteogenic BMPs (Fig. 2*D*). Given that TAPT1 attenuated BMP signaling *in vivo* as well as *in vitro* and reduced the protein levels of total SMAD1/5/9, we speculated that TAPT1 might regulate SMAD1/5/9 protein stability. To test this hypothesis, Flag-tagged SMAD1 was expressed with different amounts of TAPT1 in HEK293T cells. We found that SMAD1 protein levels were reduced by TAPT1 in a dose-dependent manner (Fig. 2*E*). Likewise, Flag-tagged SMAD5 was also downregulated by TAPT1 (Fig. 2*F*). Consistently, the endogenous protein levels of SMAD1/5/9 or SMAD1 were reduced by TAPT1, and this manner is dose-dependent (Fig. 2*G*).

We next addressed whether the TAPT1-mediated regulation of SMAD1/5 protein stability requires SMAD1/5 phosphorylation following BMP signals. A SMAD1 SEVE mutant (the C-terminal SVS phosphorylation site of SMAD1 mutates into EVE, which allows it to be independent of the BMPR signaling and acts as a constitutively active phospho-mimetic form) and a SMAD1 SAVA mutant (the C-terminal SVS phosphorylation sites were mutated into AVA, which is resistant to BMPR signaling and acts as a phosphorylation-resistant form) were introduced into HEK293T cells with TAPT1 (10, 24, 25, 26). Similar to WT SMAD1 (SWT), the protein levels of both mutants were reduced under TAPT1 overexpression (Fig. 2*H*), suggesting that the downregulatory effect of TAPT1 on SMAD1 expression likely occurs independently of BMP activity. Collectively, these results suggested that TAPT1 inhibits BMP signaling *in vivo* as well as *in vitro* and reduces SMAD1/5 protein stability.

### TAPT1 interacts with SMAD1/5, and BMP treatment increases their association

**Figure 3:**
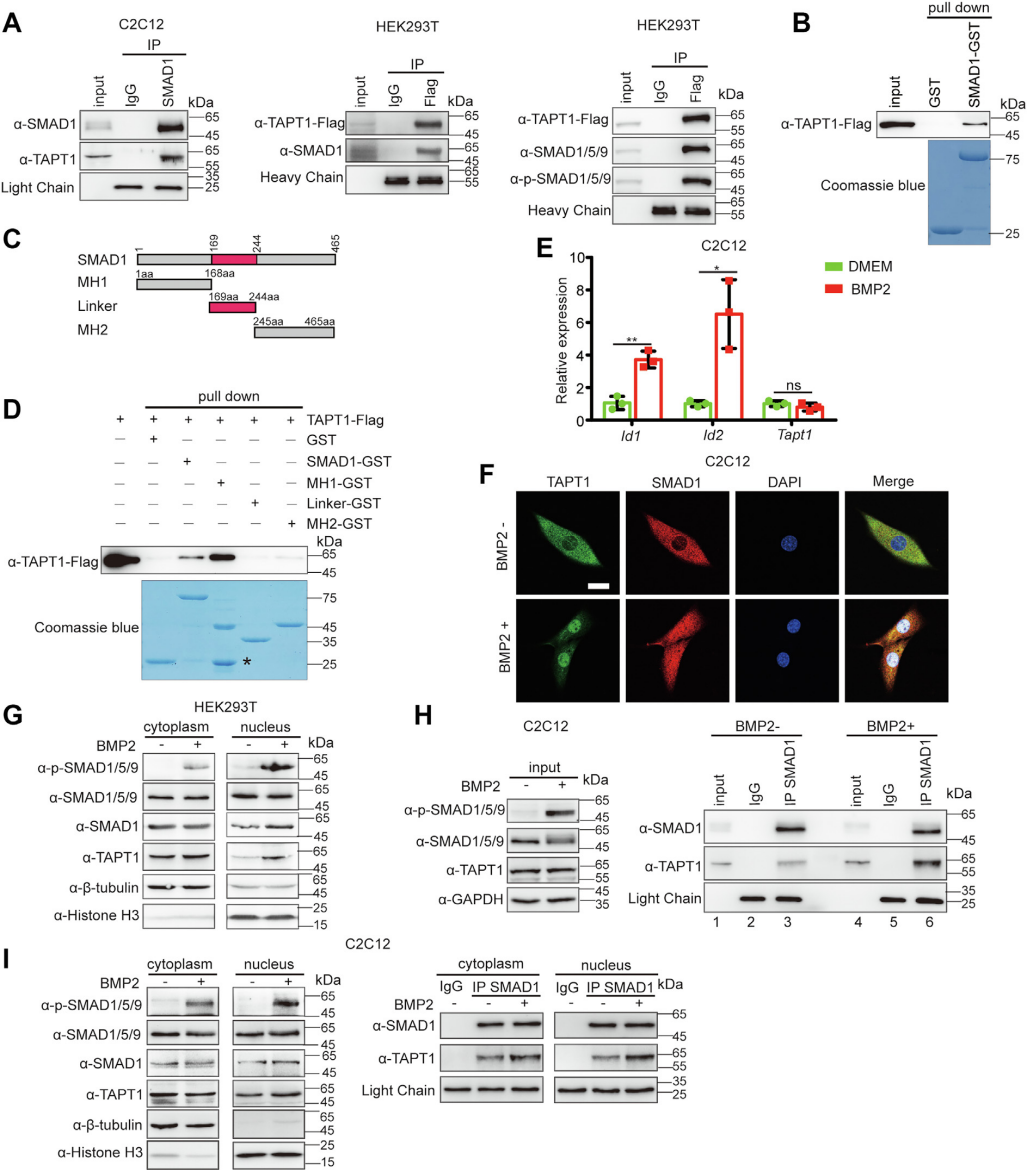
**TAPT1 associates with SMAD1, and BMP treatment increases their association.***A*, TAPT1 interacts with SMAD1/5/9, as indicated by coimmunoprecipitation. *Left panel*: endogenous SMAD1 interacts with endogenous TAPT1 in C2C12 cells. *Middle panel*: exogenous TAPT1 and endogenous SMAD1 interacted with each other in HEK293T cells. *Right panel*: exogenous TAPT1 and endogenous SMAD1/5/9 or p-SMAD1/5/9 interacted with each other in HEK293T cells. *B*, SMAD1 directly binds to TAPT1. GST and GST-SMAD1 proteins expressed by bacteria were incubated with the cell lysates from HEK293T cells transfected with Flag-TAPT1. *C* and *D*, detection of the domain in SMAD1 responsible for the TAPT1 interaction. Schematic diagram of SMAD1 protein domains is shown in (*C*). GST, GST-SMAD1, and GST-SMAD1 mutants expressed by bacteria were incubated with the extracts from HEK293T cells transfected with Flag-TAPT1. *Asterisk* indicates the nonspecific band. *E*, the *Tapt1* transcript levels in response to BMP2 stimulation. qRT-PCR was used to detect the transcription levels of *Id1*, *Id2*, and *Tapt1* with or without BMP2 treatment. *F*,distribution of endogenous TAPT1 and SMAD1 in C2C12 cells with or without BMP2 treatment. Cells were starved with serum-free medium for 1 h and subsequently stimulated with or without 100 ng/ml BMP2 protein for another 1 h. Cells were fixed and immunostained with indicated antibodies. Nuclei were labeled with DAPI. The scale bar represents 20 μm. *G*, distribution of endogenous TAPT1 and SMAD1 in the cytoplasm and nucleus of HEK293T cells with or without BMP2 treatment. *H*, the association between endogenous SMAD1 and TAPT1 in C2C12 cells with or without BMP2 treatment. C2C12 cells were starved with serum-free medium for 1 h and subsequently stimulated with or without 100 ng/ml BMP2 protein for another 1 h. An antibody against SMAD1 was used for immunoprecipitation. *I*, the interaction of endogenous TAPT1 and SMAD1 in the separated cytoplasm and nucleus of C2C12 cells with or without BMP2 treatment. The separated cytoplasm and nucleus from C2C12 cells with or without BMP2 treatment were immunoprecipitated with an anti-SMAD1 antibody. The immunoprecipitates and the inputs were analyzed by Western blotting with indicated antibodies. Similar results were obtained from three experiments. Values are means ± S.D. ∗*p* < 0.05; ∗∗*p* < 0.01; ns, not significant. Unpaired *t* test, two-tailed. BMP, bone morphogenetic protein; TAPT1, Transmembrane anterior posterior transformation 1.

To elucidate the molecular mechanism underlying the downregulation of SMAD1/5 protein stability by TAPT1, the interaction between TAPT1 and SMAD1/5/9 was assessed by coimmunoprecipitation (co-IP) assays. Endogenous TAPT1 and SMAD1 were observed in a same complex in C2C12 cells (Fig. 3*A*, left panel). Likewise, when Flag-tagged TAPT1 was expressed in HEK293T cells, endogenous SMAD1, SMAD1/5/9, and p-SMAD1/5/9 were specifically retrieved by Flag-tagged immunoprecipitates (Fig. 3*A*, middle and right panels). To further test the binding of TAPT1 and SMAD1, we conducted a pull-down assay to investigate whether the two directly bind with each other. TAPT1 directly bound to purified GST-tagged SMAD1 (Fig. 3*B*). SMAD1 contains a conserved N-terminal MH1 and C-terminal MH2 domain as well as a variable linker region (Fig. 3*C*) (6, 27). Domain mapping analysis indicated that TAPT1 bound to the MH1 domain of SMAD1 rather than to the linker or MH2 domain (Fig. 3*D*). Collectively, these results suggested that TAPT1 interacts with SMAD1.

We wondered whether the transcription of *TAPT1* is in response to BMP action. BMP2 stimulation did in fact significantly upregulate the expression of BMP target genes *Inhibitor of Differentiation 1* and *2* (*Id1* and *Id2*), respectively; however, it did not alter the mRNA levels of *Tapt1* (Fig. 3*E*). These results implied that BMP activation has little effect on the transcription of *Tapt1*. Next, we performed immunostaining with antibodies against TAPT1 and SMAD1 to observe their distribution in C2C12 cells under BMP treatment. TAPT1 and SMAD1 were mainly distributed in the cytoplasm with only partial colocalization (Fig. 3*F*). Under BMP2 stimulation, SMAD1 accumulated within the nucleus, and TAPT1 was simultaneously redistributed and translocated into the nucleus. Additionally, we noted that the colocalized signals of TAPT1 and SMAD1 increased significantly in both the cytoplasm and nucleus (Fig. 3*F*). To further confirm these results, we performed nucleocytoplasmic separation experiments to observe the distribution of TAPT1 in cells with or without BMP2 treatment. Consistently, as the p-SMAD1/5/9 accumulated in the nucleus, the amount of TAPT1 in the nucleus also increased upon BMP2 stimulation (Fig. 3*G*). Moreover, we tested whether the interaction between TAPT1 and SMAD1 was increased by a co-IP assay using C2C12 cells with or without BMP2 treatment. Treatment with BMP2 led to the activation of BMP signaling and increased p-SMAD1/5/9 levels. However, it did not change the protein levels of TAPT1 (Fig. 3*H*, left panel). The association between TAPT1 and SMAD1 increased under BMP2 treatment (Fig. 3*H*, right panel, compare lanes 3 and 6). Furthermore, we separated cell lysates into cytosolic and nuclear fractions and again performed a co-IP analysis. We found that the association between TAPT1 and SMAD1 increased both in the cytoplasm and nucleus under BMP2 treatment (Fig. 3*I*). These findings suggested that TAPT1 associates with SMAD1, with BMP signaling activation promoting their association both in cytoplasm and in the nucleus.

### TAPT1 binds to SMURF1 and promotes SMAD1 proteasomal degradation

**Figure 4:**
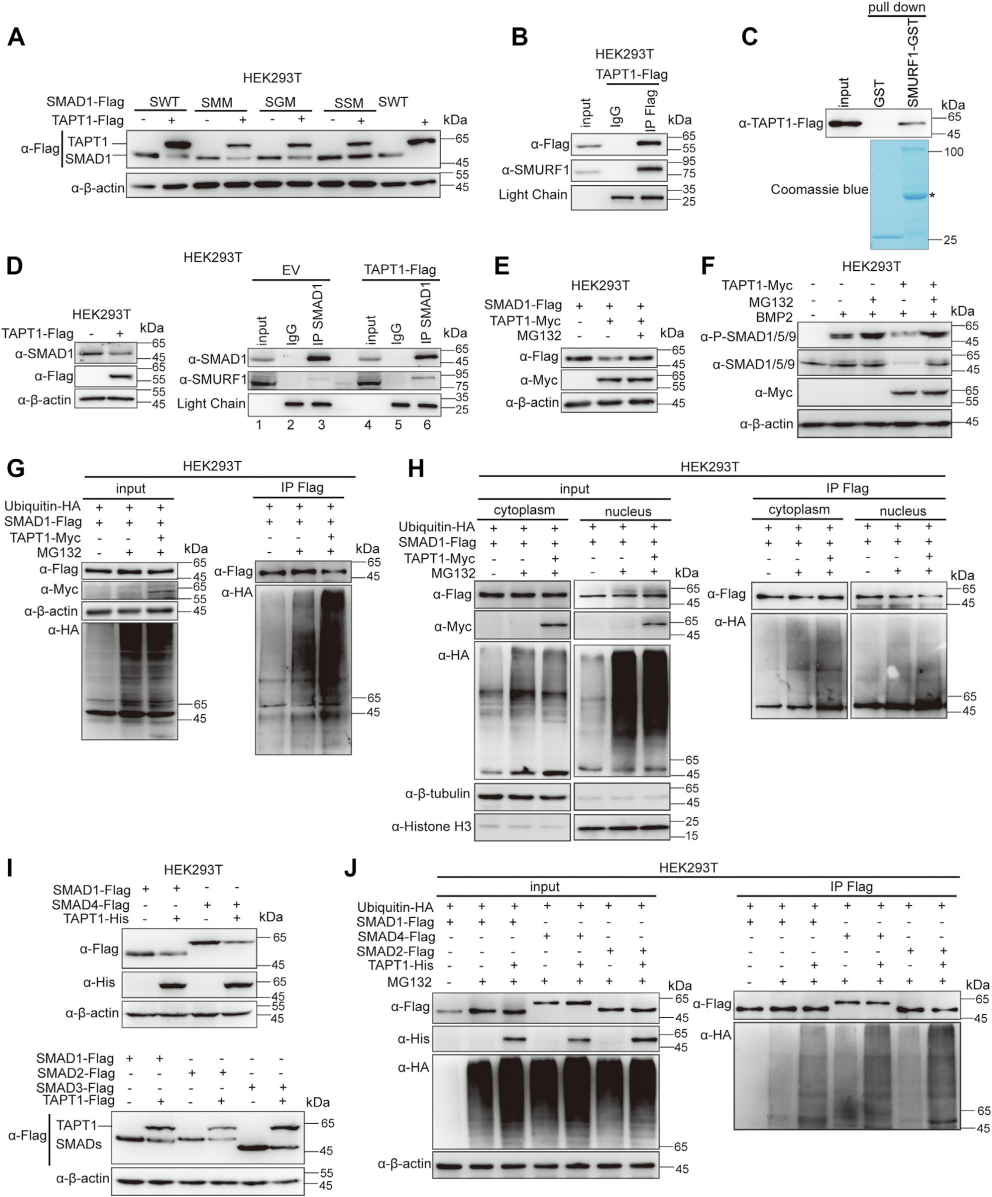
**TAPT1 promotes SMAD1 proteasomal degradation.***A*, the protein levels of WT SMAD1, SMM mutant, SGM mutant, and SSM mutant in control or TAPT1-overexpressing HEK293T cells. SMAD1 constructs encode WT SMAD1 (SWT), phosphorylation-resistant mutants for MAPK (SMM), or GSK3 (SGM) sites, and ubiquitination-resistant mutants for SMURF1 (SSM) were transfected into HEK293T cells with Flag-TAPT1 or an empty vector. *B*, exogenous TAPT1 interacts with endogenous SMURF1 as indicated by coimmunoprecipitation. *C*, SMURF1 directly binds to TAPT1. GST and GST-SMURF1 proteins expressed by bacteria were incubated with the extracts from HEK293T cells transfected with Flag-TAPT1. *Asterisk* indicates the nonspecific band. *D*, the association between endogenous SMAD1 and SMURF1 in control or TAPT1-overexpressing HEK293T cells. *E*, changes in exogenous SMAD1 protein levels in TAPT1-overexpressing HEK293T cells treated with MG132. *F*, changes of endogenous p-SMAD1/5/9 and SMAD1/5/9 protein levels in TAPT1-expressing HEK293T cells treated with MG132. Cells transfected with indicated plasmid DNA were treated with or without 10 μM MG132 for 8 h. *G*, ubiquitylation assays in HEK293T cells transfected with indicated plasmids. Cells transfected with indicated plasmid DNA and treated with or without 10 μM MG132 for 8 h. *H*, ubiquitylation assays in cytoplasm and nucleus of HEK293T cells transfected with indicated plasmids. Cells transfected with indicated plasmid DNA were treated with or without 10 μM MG132 for 8 h. The cells were then separated into cytoplasm and nucleus, followed by ubiquitination analysis. *I*, the exogenous protein levels of SMAD1, SMAD4, SMAD2, and SMAD3 in TAPT1-overexpressing HEK293T cells. The indicated plasmid was transfected into HEK293T cells with Flag-TAPT1 or an empty vector. *J*, ubiquitylation assays in HEK293T cells transfected with indicated plasmids. Cells transfected with indicated plasmid DNA were treated with or without 10 μM MG132 for 8 h. Similar results were obtained from three experiments. TAPT1, Transmembrane anterior posterior transformation 1; SMURF1, SMAD ubiquitin regulatory factor 1.

The stability of R-SMADs is critical for the activity of BMP signaling. SMAD1 interacts with, and is regulated by, multiple E3 ubiquitin ligases, such as SMURF1/2 and CHIP, to influence the strength and duration of BMP signaling (28, 29, 30). Among them, SMURF1 is the most well-characterized E3 ubiquitin ligase. SMAD1 undergoes negative regulation *via* phosphorylation at the linker region (10, 11). MAPKs (ERK, p38, and JNK) phosphorylate the linker region of SMAD1. MAPK-phosphorylated SMAD1 is recognized by GSK3, which sequentially phosphorylates the linker region (10, 11). The MAPK- and/or GSK3-phosphorylated SMAD1 is recognized by SMURF1, which leads to SMAD1 polyubiquitination and degradation in proteasome (10, 11). To investigate the molecular mechanism underlying SMAD1/5 degradation by TAPT1, we utilized a series of SMAD1 mutants, including mutants resistant to phosphorylation by MAPK (SMM) or GSK3 (SGM) and a ubiquitination-resistant mutant for SMURF1 (SSM), to assess the effect of TAPT1 on the degradation of each SMAD1 mutant. Similar to WT SMAD1 (SWT), the protein levels of SMM and SGM mutants were markedly reduced by TAPT1. In contrast, the protein levels of SSM were not altered (Fig. 4*A*). These results suggested that TAPT1 likely downregulates SMAD1 *via* proteasomal degradation. We assessed whether TAPT1 and SMURF1 formed a complex using a co-IP assay. Indeed, endogenous SMURF1 was retrieved by Flag-tagged immunoprecipitates in HEK293T cells when Flag-tagged TAPT1 was overexpressed (Fig. 4*B*). The GST pull-down assay revealed SMAD1 binds directly to SMURF1 (Fig. 4*C*). Interestingly, we observed that overexpression of Flag-tagged TAPT1 increased the association of endogenous SMURF1 and SMAD1 in HEK293T cells (Fig. 4*D*, right panel, compare lanes 3 and 6). The addition of the proteasome inhibitor MG132 resorted SMAD1 levels under TAPT1 overexpression, indicating that TAPT1-mediated SMAD1 degradation occurred *via* the proteasomal pathway (Fig. 4*E*). Moreover, addition of MG132 restored protein levels of both total SMAD1/5/9 and p-SMAD1/5/9 in TAPT1-overexpressing cells under BMP2 stimulation (Fig. 4*F*). Additionally, we examined the ubiquitylation levels of SMAD1 under TAPT1 overexpression. HEK293T cells were cotransfected with Flag-SMAD1 and Myc-TAPT1 simultaneously, thereafter subjected to ubiquitylation analysis *via* co-IP. As expected, TAPT1 dramatically enhanced the ubiquitylation of SMAD1 (Fig. 4*G*). We wondered whether the ubiquitylation of SMAD1 promoted by TAPT1 occurs in the cytosol or in the nucleus. The ubiquitination of SMAD1 was further analyzed with separated cytosolic and nuclear fractions. TAPT1 promoted the ubiquitination of SMAD1 in both cytoplasm and the nucleus (Fig. 4*H*). Taken together, these results suggested that TAPT1 binds to SMAD1 as well as to SMURF1 and promotes SMAD1 degradation *via* the proteasomal pathway in the cytoplasm and nucleus.

To address whether TAPT1 specifically regulates BMP-related SMADs and to determine the effect of TAPT1 on other SMADs, such as TGF-β–related SMADs and Co-SMAD SMAD4, we detected protein levels of SMAD4 and TGF-β–related SMAD2 and SMAD3, respectively, under TAPT1 overexpression. When TAPT1 was coexpressed with SMAD4, SMAD2, or SMAD3, TAPT1 significantly reduced protein levels of each (Fig. 4*I*). In addition, we also measured the ubiquitination levels of SMAD4 and SMAD2. When TAPT1 was coexpressed, the ubiquitination levels of SMAD4 or SMAD2 were significantly increased (Fig. 4*J*). These results suggested that TAPT1 promotes ubiquitination and proteasomal degradation of multiple SMAD proteins.

### Deficiency of TAPT1 increases SMAD1/5/9 protein levels, and TAPT1 influences the association between SMAD1 and SMURF1

**Figure 5:**
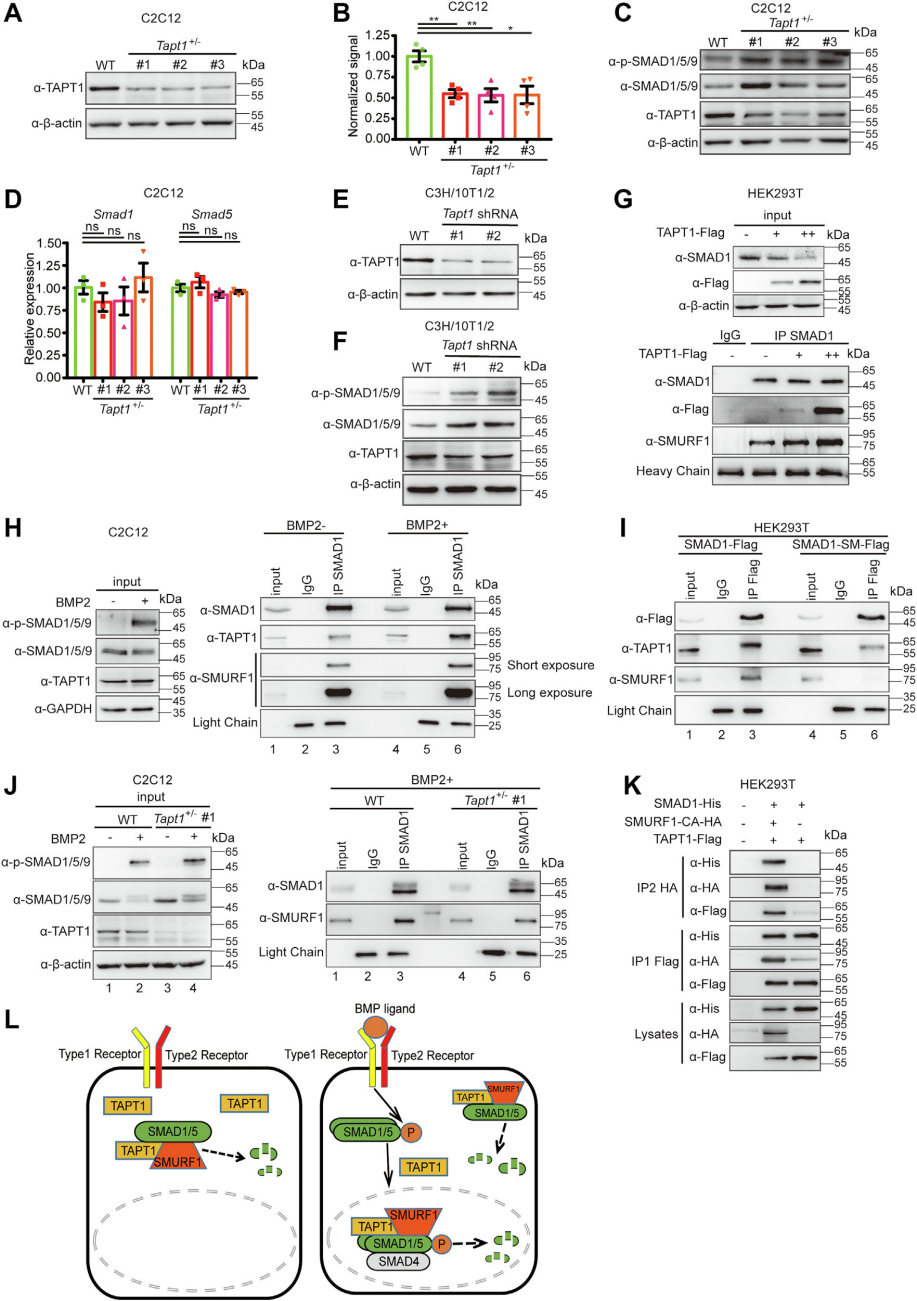
**Deficiency of TAPT1 upregulates SMAD1/5/9 protein levels, and TAPT1 promotes the interaction between SMAD1 and SMURF1.***A*, representative immunoblot and (*B*) quantification of TAPT1 protein levels in WT or indicated heterozygous TAPT1 C2C12 cells. *C*, the protein levels of p-SMAD1/5/9 and SMAD1/5/9 in WT or *Tapt1*^+/−^ C2C12 cells. *D*, qRT-PCR analysis of *Smad1* and *Smad5* transcript levels in WT and *Tapt1*^+/−^ C2C12 cells. *E*, the protein levels of TAPT1 in WT or stably TAPT1-depleted C3H/10T1/2 cells. *F*, the protein levels of p-SMAD1/5/9 and SMAD1/5/9 protein levels in WT or TAPT1-depleted C3H/10T1/2 cells. *G*, TAPT1 increases the association between endogenous SMAD1 and SMURF1 in a dose-dependent manner. TAPT1 was transfected into HEK293T cells at various doses. Proteins were extracted from cell lysates, immunoprecipitated, and subjected to Western blotting with indicated antibodies. *H*, the interaction among endogenous SMAD1, SMURF1, and TAPT1 in C2C12 cells with or without BMP2 treatment. C2C12 cells were starved for 1 h and subsequently treated with or without 100 ng/ml BMP2 protein for another 1 h. The cells were harvested, and proteins were extracted from cell lysates and then subjected to immunoprecipitation. *I*, the SMURF1 binding-resistant SMAD1 mutant (SMAD1-SM) dampened the association of SMAD1 and TAPT1 in HEK293T cells. Flag-SMAD1 or Flag-SMAD1-SM was transfected into HEK293T cells. The cells were harvested and proteins were extracted from cell lysates and then subjected to immunoprecipitation. *J*, the association between SMAD1 and SMURF1 in control or TAPT1-deficient C2C12 cells under BMP2 treatment. WT and TAPT1-deficient C2C12 cells were starved for 1 h and subsequently treated with or without 100 ng/ml BMP2 protein for another 1 h. The cell extracts were subjected to immunoprecipitation with an anti-SMAD1 antibody and Western blotting with indicated antibodies. *K*, the interaction among exogenous SMAD1, SMURF1-CA, and TAPT1. The indicated plasmids were transfected into HEK293T cells and two-step IP was performed sequentially with indicated antibodies. *L*, working model for TAPT1-regulated SMAD protein degradation. Similar results were obtained from three experiments. Data are from at least three independent experiments, with individual data points shown. Values are presented as means ± S.D. ∗*p* < 0.05; ∗∗*p* < 0.01, ns, not significant. Unpaired *t* test, two-tailed. BMP, bone morphogenetic protein; SMURF1, SMAD ubiquitin regulatory factor 1; TAPT1, Transmembrane anterior posterior transformation 1.

To further explore the effect of TAPT1 on SMAD1/5 protein degradation, *Tapt1*-deficient C2C12 cells were generated using the CRISPR/Cas9-based KO system (Fig. S2*A*). Single cells were isolated and expanded into clones that were subjected to Western blot analysis and subsequently genotyped to identify *Tapt1*-null clones. However, we did not obtain any *Tapt1*-null clones as opposed to *Tapt1* heterozygous clones (Fig. S2, *B*–*C*). Western blot analysis indicated that protein levels of TAPT1 were markedly reduced in these *Tapt1* heterozygous clones (Fig. 5, *A* and *B*). We then examined the protein levels of endogenous p-SMAD1/5/9 and total SMAD1/5/9 in these *Tapt1* heterozygous clones. Both were markedly increased in *Tapt1*^+/−^ cell lines, suggesting that *Tapt1* haploinsufficiency leads to the upregulation of p-SMAD1/5/9 and total SMAD1/5/9 (Fig. 5*C*). Besides, *Smad1* and *Smad5* expression did not change based on qRT-PCR analysis (Fig. 5*D*). Similar results were also observed in stably shRNA-mediated TAPT1-knockdown C3H/10T1/2 cells (Fig. 5, *E* and *F*). Taken together, these results suggested that TAPT1 downregulation increases endogenous SMAD1/5/9 protein levels.

As mentioned above, overexpression of TAPT1 increased the association between SMAD1 and SMURF1. This may facilitate SMAD1 proteasomal degradation. To test this hypothesis, we performed a co-IP assay using HEK293T cells with increased expression of TAPT1. We found that the amounts of endogenous SMURF1 or exogenous Flag-tagged TAPT1 that coprecipitated with endogenous SMAD1 increased with greater doses of TAPT1 (Fig. 5*G*). Additionally, treatment of C2C12 cells with BMP2 simultaneously increased the association of endogenous TAPT1 with SMAD1 as well as that of endogenous SMURF1 with SMAD1 (Fig. 5*H*, right panel, compare lanes 3 and 6). These results suggested that the increased binding of SMURF1 to SMAD1 and TAPT1 to SMAD1 occurred concurrently. We then examined whether binding between TAPT1 and SMAD1 required SMURF1. Indeed, when the SMURF1-binding sites on SMAD1 were mutated and SMURF1 no longer bound to SMAD1, the TAPT1 and SMAD1 interaction was significantly suppressed (Fig. 5*I*, compare lanes 3 and 6). These results prompted us to speculate that TAPT1 facilitates the binding of SMAD1 and SMURF1 to promote SMAD1 proteasomal degradation. To test this hypothesis, we utilized *Tapt1* heterozygous cells under BMP treatment to examine whether the association between SMAD1 and SMURF1 decreased in the *Tapt1-*reduced condition, as it is easier to observe the alteration of association between endogenous SMURF1 and SMAD1 under BMP treatment. Indeed, reduction of TAPT1 upregulated the protein levels of p-SMAD1/5/9 and total SMAD1/5/9 (Fig. 5*J*, left panel, compare lanes 2 and 4). As expected, the binding of SMURF1 to SMAD1 was decreased in *Tapt1* heterozygous cells in the presence of BMP2 stimulation, suggesting that insufficient TAPT1 reduces the association between SMAD1 and SMURF1 (Fig. 5*J*, right panel, compare lanes 3 and 6), which in turn increases the protein levels of p-SMAD1/5/9 and total SMAD1/5/9. The above results suggested that TAPT1, SMURF1, and SMAD1 are likely in a same complex. We next tested this possibility with a two-step IP assay (31). As expected, SMAD1 was detected only when all of TAPT1, SMURF1, and SMAD1 were coexpressed (Fig. 5*K*), suggesting that TAPT1 forms a ternary complex with SMURF1 and SMAD1. Taken together, we propose a role for TAPT1 in BMP signaling: TAPT1 facilitates the binding of SMURF1 and SMAD1/5, thereby promoting the proteasomal degradation of SMAD1/5 in both the cytoplasm and the nucleus; the association of TAPT1 with SMAD1/5 is increased under BMP2 treatment (Fig. 5*L*).

### Deficiency of TAPT1 boosts transdifferentiation of C2C12 myoblasts and differentiation of C3H/10T1/2 mesenchymal stem cells into mature osteoblasts

**Figure 6:**
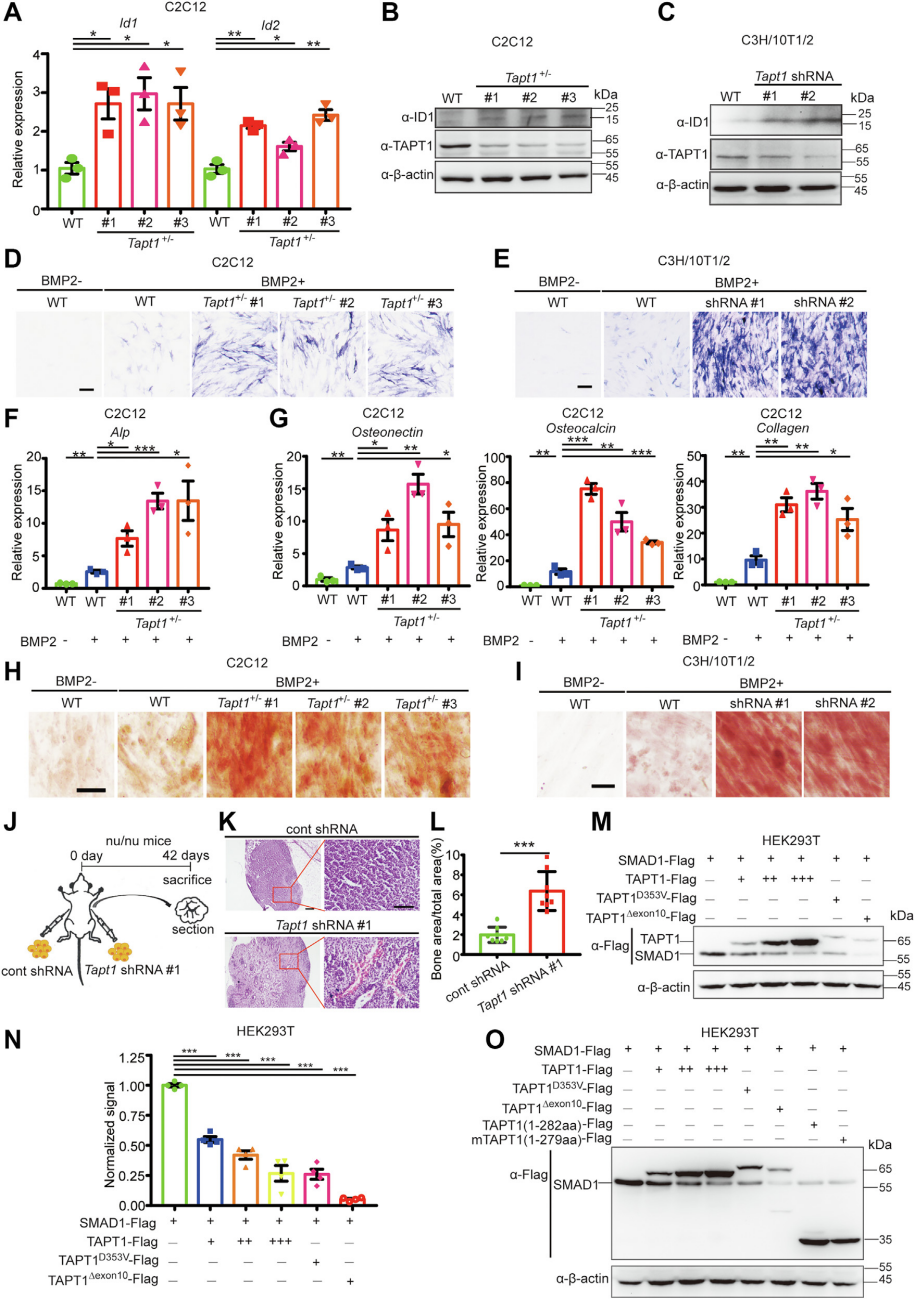
**Deficiency of TAPT1 in C2C12 myoblasts or C3H/10T1/2 mesenchymal stem cells boosts their transdifferentiation/differentiation toward a mature osteoblast fate under BMP treatment.***A*, the mRNA levels of BMP target genes *Id1* and *Id2* in WT or *Tapt1*^*+/−*^ C2C12 cells were analyzed by qRT-PCR. *B* and *C*, ID1 protein levels in WT or TAPT1-deficient C2C12 or C3H/10T1/2 cells. *D*, representative images of osteogenic marker alkaline phosphatase (ALP) activity in transdifferentiated WT and *Tapt1*^*+/−*^ C2C12 myoblasts under BMP treatment. WT and *Tapt1*^*+/−*^ C2C12 myoblasts were treated with BMP2 for 6 days. Media were changed every 2 days. After 7 days incubation, the cells were stained with NBT/BCIP. *E*, representative images of osteogenic marker ALP activity in differentiated WT and TAPT1-deficient C3H/10T1/2 cells under BMP treatment. *F*, quantification of the transcriptional levels of *Alp* in WT or *Tapt1*^*+/−*^ C2C12 cells with BMP2 treatment by qRT-PCR. *G*, the transcriptional levels of mature osteoblast markers, *Osteonectin*, *Osteocalcin*, and *Collagen*, in WT or *Tapt1*^*+/−*^ C2C12 cells with BMP2 treatment were analyzed by qRT-PCR. *H* and *I*, representative images of alizarin red S staining of the mature osteoblasts in WT or TAPT1-deficient C2C12 (*H*) or C3H/10T1/2 cells (I) with BMP2 treatment. C2C12 myoblasts or C3H/10T1/2 cells were treated with BMP2 for 21 days. Media were changed every 2 days. After 21 days of incubation, the cells were stained with alizarin red S. The scale bar represents 200 μm. *J*, schematic depicting the establishment of an ectopic bone formation model. *K*, representative images of osteogenic differentiation of the WT or *Tapt1* shRNA C3H/10T1/2 cells in mice. WT and TAPT1-depleted C3H/10T1/2 cells were transplanted into nude mice. After 42 days, the mice were sacrificed to remove the transplants. The transplants were then sectioned and stained with H&E. The scale bar represents 100 μm. *L*, quantitative results of bone area in (*K*). n = 8. *M*, the protein levels of Flag-SMAD1 in control or various doses of WT TAPT1-, TAPT1^D353V^ mutant-, or TAPT1^Δexon10^ mutant-overexpressing HEK293T cells. Each indicated plasmid with various doses or an empty vector was transfected into HEK293T cells with Flag-SMAD1. *N*, quantification of Flag-SMAD1 expression levels shown in (*M*). Similar results were obtained from four experiments. Data are from four independent experiments with individual data points shown. *O*, the protein levels of Flag-SMAD1 in control, or WT TAPT1-or truncated TAPT1 mutant-overexpressing HEK293T cells. Each indicated plasmid with various doses or an empty vector was transfected into HEK293T cells with Flag-SMAD1. Data are from at least three independent experiments, with individual data points shown. Values are presented as means ± S.D. ∗*p* < 0.05; ∗∗*p* < 0.01, ∗∗∗*p* < 0.001, Unpaired *t* test, two-tailed. BMP, bone morphogenetic protein; TAPT1, Transmembrane anterior posterior transformation 1.

The activation of BMP signaling in C2C12 myoblasts increases the transcription of BMP target genes *Id1* and *Id2* (Fig. 3*E*) (32). Therefore, we examined the transcriptional levels of *Id1* and *Id2* in *Tapt1*^+/−^ C2C12 myoblasts. As shown in Figure 6*A*, the mRNA levels of *Id1* and *Id2* were significantly increased in *Tapt1*^+/−^ C2C12 cells. Consistently, ID1 protein levels were also increased in *Tapt1*^+/−^ C2C12 myoblasts (Fig. 6*B*). Similar result was observed in TAPT1-knockdown C3H/10T1/2 cells (Fig. 6*C*). These results suggested that depletion of TAPT1 leads to the induction of BMP target genes. The activation of BMP signals is key to driving bone formation. Previous studies have indicated that BMP treatment can induce the osteoblastic transdifferentiation of C2C12 myoblasts or differentiation of C3H/10T1/2 mesenchymal stem cells (33, 34, 35). Hence, TAPT1-deficient C2C12 cells or C3H/10T1/2 cells were used to examine whether TAPT1 insufficiency increased the cellular response to BMP. WT and *Tapt1*^+/−^ cells were stimulated with 300 ng/ml BMP2 recombinant protein. After 6 days of culture, the cells were fixed and stained for alkaline phosphatase (ALP), an early osteoblastic differentiation marker. WT cells exhibited a very limited number of ALP-expressing cells, while strong ALP expression was observed in *Tapt1*^+/−^ C2C12 cells (Fig. 6*D*). Similar results were observed in stably TAPT1-depleted C3H/10T1/2 cells (Fig. 6*E*). In addition, we examined *Alp* transcript levels in TAPT1-deficient cells. As expected, *Tapt1*^+/−^ cells with BMP2 treatment led to a robust induction of *Alp* mRNA levels, while only a slight induction occurred in the BMP2-treated WT cells (Fig. 6*F*). We also monitored changes in the gene expression levels of other osteoblast markers, including *Osteonectin*, *Osteocalcin*, and *Collagen*, in BMP2-treated WT and *Tapt1*^+/−^ cells, by qRT-PCR analysis. Treatment with BMP2 only slightly induced the expression of these marker genes in WT cells. In contrast, *Tapt1*^+/−^ cells treated with BMP2 exhibited a marked induction of osteoblast markers (Fig. 6*G*). To further confirm that insufficient TAPT1 leads to enhanced sensitivity to BMP, we also examined the degree of mineralization to evaluate the formation of mature osteoblasts in WT and TAPT1-deficient cells under BMP2 treatment. Cells were treated with 300 ng/ml BMP2 for 22 days and then mineralization was detected by staining with alizarin red S solution. The WT cells treated with 300 ng/ml BMP2 showed slight mineralization, while the *Tapt1*^+/−^ cells treated with 300 ng/ml BMP2 exhibited strong mineralization (Fig. 6*H*). Likewise, TAPT1-depleted C3H/10T1/2 cells had similar responsiveness upon BMP2 treatment (Fig. 6*I*). To confirm these results, we evaluated the inhibitory effect of TAPT1 on osteogenic differentiation *in vivo* using ectopic bone formation in mice. In the transplants of stably TAPT1-depleted C3H/10T1/2 cells in athymic nude (nu/nu) mice, the degree of mineralization was increased significantly when compared with control group (Fig. 6, *J*–*L*). Altogether, these results suggest that *Tapt1* insufficiency boosts both transdifferentiation of C2C12 myoblasts and differentiation of C3H/10T1/2 mesenchymal stem cells into mature osteoblasts.

Two homozygous TAPT1 mutations, *TAPT1*^*D353V*^ and *TAPT1*^*Δexon10*^, cause a congenital syndrome with complex lethal osteochondrodysplasia, showing severe hypomineralization of the entire skeleton (Fig. S1*A*) (14). We therefore speculated that these two mutants may alter the effects of TAPT1 on the activity of BMP signaling. We noticed that even low levels of the Flag-tagged TAPT1 mutants downregulated SMAD1 dramatically when compared to WT TAPT1 (Fig. 6, *M* and *N*). These results suggest that TAPT1^D353V^ and TAPT1^Δexon10^ likely exhibit GOF activities in the regulation of BMP signaling. In addition, ethylnitrosourea-induced mutations in the mouse *Tapt1* gene resulted in a truncated form of TAPT1, TAPT1 (1–279), which is encoded only by exons 1 to 6 and causes homeotic-like skeletal transformations (Fig. S1*A*) (12). Next, we determined the effects of mouse TAPT1(1–279) and human TAPT1(1–282) (a truncated form of human TAPT1 that corresponds to mouse TAPT1 (1–279)) on the protein levels of SMAD1. Like WT TAPT1, both mutants downregulated the protein levels of SMAD1 (Fig. 6*O*). These results suggested that the truncated mutants have comparable effects on BMP signaling.

## Discussion

In the present study, we uncovered a mechanism by which TAPT1 functions as a novel binding partner of SMAD1 and SMURF1 to form a ternary complex and inhibits the BMP signaling pathway. We found that TAPT1 facilitates the association of SMAD1 and SMURF1, which in turn promotes the proteasomal degradation of SMAD1 in the cytoplasm and the nucleus. In addition, TAPT1 promoted the association of SMURF1 and SMAD1 in response to BMP2 stimulation. TAPT1 deficiency promoted ossification of C2C12 and C3H/10T1/2 cells by augmenting the BMP signaling pathway. Importantly, two congenital syndrome-causing mutations of *TAPT1* exhibited GOF activities in the regulation of BMP signaling. In the zebrafish embryos, we observed that TAPT1/Tapt1 attenuated Bmp signaling to promote dorsal development.

R-SMADs in BMP signaling are regulated at multiple levels from the cytoplasm to the nucleus. Similar to many signal transducers, the activity of R-SMADs is tightly controlled, particularly through posttranslational modifications, including phosphorylation and ubiquitination (36). Notably, the duration of SMAD1 activation is mainly regulated *via* polyubiquitinylation at the conserved SMURF1 recognition sites within the linker region (30). In cells with basal BMP levels, SMURF1 targets SMAD1/5 in the cytoplasm for destruction (30). This function may be important for maintaining the basal state of unstimulated cells. In addition, various unknown aspects of SMURF1 function remain unexplored, including how its activity is regulated and how substrates are selected. Many proteins are involved in SMAD1/5 activity through the regulation of SMURF1. For example, CKIP-1 specifically targets the linker region of SMURF1 and augments its association with SMAD5, thereby promoting ubiquitylation of the latter (37).

Although the interaction between SMAD5 and SMURF1 is regulated by CKIP-1, there is no direct interaction between CKIP-1 and SMAD5 in the absence of SMURF1 (37). Our results on TAPT1 differ from those previous studies on CKIP-1. We found that TAPT1 binds with SMURF1 and with SMAD1 to form a ternary complex. Since BMP2 treatment increases the association between SMAD1 and TAPT1 and TAPT1 promotes ubiquitination of SMAD1 both in the cytoplasm and in the nucleus, it is likely that the formation of a ternary complex with these three occurs both in the cytoplasm and in the nucleus. In this complex, TAPT1 facilitates the degradation of SMAD1 by promoting the interaction between SMURF1 and SMAD1. Several lines of evidence support our findings. The evidence includes the following: 1) TAPT1 directly interacts with SMAD1 and SMURF1 as well as these three forms a complex; 2) TAPT1 overexpression promotes the interaction between SMAD1 and SMURF1; 3) TAPT1 deficiency weakens the interaction between SMAD1 and SMURF1; 4) the interaction between TAPT1 and SMAD1 is weakened when SMAD1 does not bind SMURF1. These suggest that TAPT1 influences the interaction between SMURF1 and SMAD1. Intriguingly, we found that the interactions of SMAD1 with TAPT1 and with SMURF1 were enhanced concurrently when the BMP signaling pathway was activated. Indeed, upon activation of BMP signaling, negative feedback mechanisms attenuate and/or terminate the signaling cascade(s) (38). Herein, we identified TAPT1 as a novel binding partner of SMAD1 and SMURF1 and highlighted its relevance in promoting the binding of SMAD1 and SMURF1 in response to BMP2 stimulation. Hence, TAPT1 likely acts as a negative regulator of BMP signaling activity by stringently regulating the stability of SMAD1/5 *via* its redistribution upon the activation of BMP signaling. Taken together, our study identified a novel inhibitor of BMP signaling and provided a possible explanation for the negative regulation of BMP signaling.

Multiple studies have suggested that SMAD1 polyubiquitylation requires linker phosphorylation to mediate negative feedback (10, 11). As mentioned earlier, R-SMAD proteins contain a DNA-binding domain, the MH1 domain, a transactivation domain, the MH2 domain, and a less conserved linker region. R-SMADs are regulated by type I receptors and are phosphorylated through the C-terminal SSXS motif of the MH2 domain. Subsequently, R-SMADs form heterotrimeric complexes with SMAD4 through the C-terminal SSXS motif. After C-terminal phosphorylation by BMPR, the linker region is phosphorylated at MAPK sites (10). Phosphorylation of GSK3 requires phosphorylated MAPK sites. The ubiquitination of SMAD1 requires MAPK and GSK3 phosphorylation in order to regulate BMP signaling (10, 11). Within the nucleus, the linker region of SMAD1 that associates with SMAD4 is also phosphorylated by CDK8/9, which creates binding sites for competing YAP transcription factor and SMURF1. The SMAD1 linker is subsequently phosphorylated by GSK3, which switches off YAP1 binding and facilitates association with SMURF1 (39). TAPT1 interacts with SMAD1 and with SMURF1, with BMP activation facilitating their association. Based on our results, we suggest the following working model of TAPT1 in complex with SMAD1 and SMURF1: once TAPT1 binds to the SMAD1 MH1 domain, the structure of SMAD1 may be altered, which allows its linker PPXY motif to be more easily recognized by the SMURF1 E3 ubiquitin ligase.

The PPXY motif is critical for binding with WW domain of SMURF2 as for binding with SMURF1 (29, 30). In addition, CHIP E3 ubiquitin ligase binds to the MH2 domain of SMAD1 and leads to ubiquitination and degradation of SMAD1 (28). Our results suggested that mutation of PPXY motif in SMAD1 is sufficient to prevent the downregulatory effect of TAPT1. This raises a question that TAPT1 may also facilitate SMURF2 associate with SMAD1 to promote SMAD1 degradation *via* the proteasomal pathway. This issue needs further investigation. The previously reported E3 ubiquitin ligases, such as SMURF2, WWP1, and NEDD4, contain the WW domain which binds to SMAD2/3 and promotes its ubiquitination and degradation (40). We also observed that TAPT1 promotes ubiquitination and degradation of SMAD2/3. Further studies will be required to elucidate the underlying molecular mechanisms of TAPT1 involved in the ubiquitination and degradation of SMAD2/3. Besides, SMURF1/2 do not directly interact with SMAD4 due to SMAD4 lacking a PPXY motif (29). The ubiquitination and degradation of SMAD4 is regulated by SMURF1/2 in the presence of R-SMADs (41). This may explain the ubiquitination and destabilization of SMAD4 under TAPT1 overexpression. Additionally, SMURF1 interact with I-SMADs, such as SMAD7, through its PPXY motif. This allows recruitment of SMURF1 to the activated TGF-β type I receptor *via* SMAD7 and which leads to ubiquitination and degradation of TGF-β type I receptor (42). Although TAPT1 binds with MH1 domain in SMAD1 and I-SMADs lack MH1 domain, we cannot exclude the possibility that TAPT1 also binds with SMAD7 and leads to the degradation of receptors and SMAD7. This issue needs to be determined in the future.

Despite TAPT1 overexpression destabilized BMP-related SMAD1/5, TGF-β/NODAL-related SMAD2/3, and SMAD4, forced expression of TAPT1 leads to dorsalization of zebrafish embryos. These dorsalized phenotypes are reminiscent of maternal-zygotic *smad5* (MZ*smad5*), while not MZ*smad2* mutants (43, 44). Interestingly, Smad4a-depleted zebrafish mutants exhibited dorsalized phenotypes with complete loss of Bmp action but normal Nodal signaling transcription profiles (45). Thus, the dorsalizing effects of TAPT1/Tapt1 likely result from the reduced activity of Smad5 and Smad4a, although dorsoventral patterning is the result of comprehensive action of these types of signaling.

The phenotypes of TAPT1 in humans and mice are diametrically different. In humans, *TAPT1* mutations show a clinical phenotype with lethal skeletal dysplasia. This syndrome is characterized by fetal lethality with severe hypomineralization of the entire skeleton as well as intrauterine fractures (14). However, nearly half of the mice-carrying mutant *Tapt1* develop a 14th pair of ribs, and, in most mutants, the xiphoid process was overgrown and splayed (12). Deficiency of multiple signaling factors or mediators in the BMP signaling pathway leads to phenotypes associated with skeletal development (46). BMP2/4 promoted the transdifferentiation of C2C12 myoblasts into osteoblasts (33, 47). Likewise, BMP2 stimulation enhanced the degree of mineralization of C3H/10T1/2 mesenchymal stem cells (34, 35). In addition, excessive activation of BMP signaling is an important cause of multiple synostoses syndrome, which is characterized by the fusion of multiple joints (48, 49). In the present study, we demonstrated that TAPT1 deficiency promoted the ossification of C2C12 myoblasts and C3H/10T1/2 mesenchymal stem cells by amplifying BMP signaling. TAPT1 deficiency leads to upregulation of BMP target genes and amplifies the response of C2C12 cells and C3H/10T1/2 cells, two distinct types of cell lineages, to BMP stimulation, promoting osteoblastic transdifferentiation or differentiation. Importantly, we also found that the two congenital syndrome–causing mutations of *TAPT1* exhibit GOF activities in the regulation of SMAD1 protein stability. These enhanced effects of TAPT1 mutations on BMP signaling are likely to cause the clinical phenotype of skeletal dysplasias (14). In mice, the ethylnitrosourea-induced *Tapt1* mutation resulted in a truncated TAPT1, which downregulated SMAD1. This may explain the phenotypic differences between the human and mice carrying the distinct mutations. Taken together, it is likely that TAPT1 functions in bone development by negatively modulating the BMP signaling pathway.

In conclusion, our study uncovered a negative regulatory role of TAPT1 in the BMP signaling pathway. Mechanistically, TAPT1 promotes the binding of SMAD1 and SMURF1 in response to BMP2 stimulation, facilitating the proteasomal degradation of SMAD1. The function of TAPT1 in BMP signaling pathway may help us to understand the mechanisms underlying the reported complex congenital syndrome caused by *TAPT1* mutation.

## Experimental procedures

### Chemicals, reagents, and antibodies

Oligo(dT)_18_ was purchased from Sangon Biotech (Shanghai). M-MLV Reverse Transcriptase was purchased from Promega. DIG-UTP and anti-digoxigenin-AP were purchased from Roche. The mMESSAGE mMACHINE mRNA Synthesis Kit was purchased from Ambion. Protein A/G Plus-agarose was purchased from Santa Cruz Biotechnology. Dulbecco’s modified Eagle’s medium and MEM was purchased from Hyclone. Fetal bovine serum (FBS) was purchased from PAN. Human BMP-2 protein was purchased from Novus.

The following antibodies were used in this study: mouse anti-SMAD1 (1:500 for Western blotting, 1:100 for immunocytochemistry, 2 μg for co-IP, SAB1404035; Sigma), rabbit anti-TAPT1 (1:500 for Western blotting, 1:100 for immunocytochemistry, 2 μg for co-IP, SAB1301658; Sigma), rabbit anti-phospho-SMAD1/5/9 (1:1000 for Western blotting; #13820; Cell Signaling Technology), rabbit anti-SMAD1/5/9 (1:500 for Western blotting; sc-6031-R; Santa Cruz Biotechnology), mouse anti-ID1 (1:500 for Western blotting; sc-133104; Santa Cruz Biotechnology), mouse anti-SMURF1 (1:500 for Western blotting; ab57573; Abcam), rabbit anti-HA (1:1000 for Western blotting and 2 μg for co-IP assays, #3724; Cell Signaling Technology), mouse anti-Myc (1:1000 for Western blotting; sc-40; Santa Cruz Biotechnology), murine anti-Flag (1:1000 for Western blotting and 2 μg for co-IP assays; F1804; Sigma), rabbit anti-His (1:1000 for Western blotting; #12698; Cell Signaling Technology), rabbit anti-GAPDH (1:4000 for Western blotting, D110016; Sangon Biotech), rabbit anti-β-tubulin (1:1000 for Western blotting; #2146; Cell Signaling Technology), rabbit anti-Histone H3.1 (1:1000 for Western blotting; P30266; Abmart), and rabbit anti-β-actin (1:1000 for Western blotting; abs132001; Absin).

### Zebrafish strains

Zebrafish (*Danio rerio*, Tübingen strain) were maintained on a 14 h light/10 h dark cycle at 28.5 °C and fed twice daily. Embryos were reared in embryo medium in an incubator at 28.5 °C. The stages of the embryos were determined according to standard methods (50). All experimental protocols were approved by and performed according to the guidelines set by the Ethical Committee of Experimental Animal Care, Ocean University of China.

### Molecular cloning and plasmid construction

The pCS2-Flag-TAPT1, pCDNA3.1-Myc-TAPT1, pCS2-GFP-TAPT1, pCS2-Flag-Tapt1a, pCS2-Flag-Tapt1b, and pCS2-Flag-SMAD1 were generated by PCR subcloning. The pCS2-Flag-SMAD1 SEVE, pCS2-Flag-SMAD1 SAVA, pCS2-Flag-SMAD1 SMM, pCS2-Flag-SMAD1 SGM, pCS2-Flag-SMAD1 SSM, and pCDNA3.1-HA-SMURF1-CA mutants were generated by site-directed mutagenesis. Briefly, the human *TAPT1* and zebrafish *tapt1a*/*tapt1b* ORFs were amplified *via* PCR from HEK293T cells or zebrafish complementary DNA. The resultant product was subcloned into different vectors, respectively. All primers for plasmid construction are listed in the Table S1. Amino acid sequence alignment was performed using ClustalX and GeneDoc.

### Quantitative real-time RT-PCR and whole-mount *in situ* hybridization

Total RNA was isolated from zebrafish embryos or cultured cells using RNAiso plus reagent (Takara Bio). Subsequently, 2 μg of the RNA template was reverse transcribed into complementary DNA using oligo(dT)_18_ and M-MLV, according to the manufacturer’s instructions. Quantitative PCR analyses were performed using the iTaq SYBR Green Supermix and iCycler apparatus (Bio-Rad Laboratories). Data were collected from at least three independent experiments, and each sample was measured in duplicate. The mRNA levels of the indicated genes were calculated as per the 2^-ΔΔCt^ method and normalized to *β-actin* or *GAPDH*.

Whole-mount *in situ* hybridization was performed as reported previously (51, 52, 53). The plasmid of *tapt1a/tapt1b*-containing partial ORF and 3′ UTR was used to synthesize the sense probe and antisense riboprobes. The specificity of riboprobes was verified *via* dot blot analysis. Image acquisition was performed using a dissecting stereomicroscope.

### Capped mRNA synthesis and microinjection

The plasmid with the desired gene is linearized and used as a template for the synthesis of capped mRNA. The mRNA is diluted to the appropriate concentration and injected into the yolk of embryos at the one-two cell stage. The injected embryos were kept in embryo-rearing medium in an incubator and maintained at 28.5 °C. Embryos were raised to the indicated stages and collected for subsequent experiments.

### Cell culture

HEK293T, C2C12, and C3H/10T1/2 cell lines were purchased from ATCC. HEK293T, C2C12, and C3H/10T1/2 cells were cultured in Dulbecco’s modified Eagle’s medium and MEM medium, respectively, supplemented with 10% FBS and 1% penicillin/streptomycin. The cells were cultured in a humidified incubator at 37 °C with 5% CO_2_. All cell lines were authenticated with short tandem repeat profiling by Shangai Cell-Bank. The culture cells were tested to be free of *mycoplasma* contamination by EZ-PCR Mycoplasmas Detection Kit (BI, Kibbutz Beit-Haemek) every 3 months. Plasmids were transfected into cells by PEI (Polysciences, cat# 23966-2) following the manufacturer’s instructions.

### Immunoblotting and immunoprecipitation

Immunoblotting and Co-IP were performed as previously described (54). In brief, cells at 70 to 80% confluence were transfected with plasmid(s). Twenty four hours after transfection, the cells were lysed with RIPA buffer (150 mM NaCl, 1% Triton X-100, 1% sodium deoxycholate, 0.1% SDS, 50 mM Tris at pH 7.5) supplemented with protease and phosphatase inhibitors. Proteins were separated by SDS-PAGE, transferred to PVDF membranes, and incubated with primary as well as secondary antibodies. β-actin, GAPDH, β-tubulin, or Histone H3 was used as a loading control. All the results were obtained from at least three independent experiments. Representative results are shown in the figures.

For Co-IP experiments, cells were lysed in IP lysis buffer (50 mM Tris at pH 7.5, 150 mM NaCl, 1 mM EDTA, 10% glycerol, 1% Triton X-100, as well as protease and phosphatase inhibitors). Cleared cell lysates were incubated with the indicated antibody and incubated with protein A/G agarose beads. Subsequently, the protein complexes bound to protein A/G beads were briefly washed and immunoprecipitates were denatured and dissolved in loading buffer by boiling the beads, followed by immunoblotting. For two-step IP, the process was performed as previously described (31). First, the cell lysates were incubated with an anti-Flag antibody and incubated with protein A/G agarose beads, followed by elution with Flag peptides. The eluent was then incubated with an anti-HA antibody and incubated with protein A/G agarose beads. These complexes bound to protein A/G beads were washed, and immunoprecipitates were eluted in loading buffer by heating the beads, followed by Western blot analysis. All the results were obtained from three independent experiments. Representative results are shown in the figures.

### Immunofluorescence staining

C2C12 or C3H/10T1/2 cells were fixed with 4% paraformaldehyde (w/v). Subsequently, the fixed cells were permeable by 0.2% Triton X-100 and blocked with 20% FBS. The cells were then incubated with appropriate primary and secondary antibodies along with DAPI to visualize nuclei.

Embryos at the indicated stages were fixed with 4% paraformaldehyde. Subsequently, they were permeated with 0.8% Triton X-100 and blocked with 20% FBS. After incubation of primary and Alexa-488– or Cy3-conjugated secondary antibodies, nuclei were then counterstained with DAPI. Image acquisition was performed using a Leica SP8 confocal microscope (Leica Microsystems).

### Ubiquitination assay

*In vivo* ubiquitination assays were performed as previously described (55). Briefly, HA-Ub, Flag-SMAD1, and Myc-TAPT1 were cotransfected in HEK293T. Cells were treated with MG132 (10 μM) for 8 h prior to harvesting, and subsequently, cells were hot-lysed by boiling in denaturing buffer (2% SDS, 150 mM NaCl, 10 mM Tris–HCl [pH 8.0]) with 2 mM N-ethylmaleimide and protease inhibitors. After adding 9 times the volume of diluent buffer (10 mM Tris–HCl [pH 8.0], 150 mM NaCl, 2 mM EDTA, 1% Triton X-100), an anti-Flag antibody and Protein A/G beads were added and incubated. The beads were then extensively washed. Ubiquitinated SMAD1 was detected *via* immunoblotting using the indicated antibodies. All the results were obtained from three independent experiments. Representative results are shown.

### Generation of TAPT1-deficient cells

CRISPR/Cas9 sgRNAs targeting the first exon region of the *Tapt1* gene were used to generate TAPT1-deficient C2C12 cell lines. Specific sgRNAs were designed by http://crispor.tefor.net/crispor.py and cloned into a CRISPR V2 plasmid (http://zifit.partners.org/). The sgRNAs plasmid and two packaging plasmids were cotransfected into HEK293T cells. Forty eight hours after transfection, the supernatant was collected and filtered through a 0.22 μm filter. C2C12 cells at 30 to 40% confluence were added with viral supernatant containing 8 μg/ml polybrene for infection. Post 48 h of infection, puromycin was added to select puromycin-resistant cells. Single knockout-positive cells were isolated in 96-well plates using a gradient dilution method. The knockout efficiency was detected *via* immunoblotting, and *Tapt1* mutation was determined by sequencing.

The stable TAPT1-knockdown C3H/10T1/2 cell lines were established by lentiviral delivery of shRNA in the C3H/10T1/2 cells. The sequences targeting *Tapt1* were designed by https://www.sigmaaldrich.cn and cloned into lentiviral pLKO.1-GFP+Puromycin vector. Sequences were as follows: shRNA#1: 5′-CATCCGAAATTGCTGTGGATA-3′; shRNA#2: 5′-GCTGTCTTACTCATCAGAGTT-3′. The lentiviral plasmid and two packaging plasmids were cotransfected into HEK293T cells. Virus was collected and infected with C3H/10T1/2 cells. Puromycin was added to the culturing medium for selection. The knockdown efficiency was detected *via* immunoblotting.

### ALP and alizarin red S staining

For ALP staining, WT or TAPT1-deficient C2C12 or C3H/10T1/2 cells were seeded in 24-well culture plates (day 0) and treated with BMP2 for 6 days. The media with BMP2 were changed every 2 days. Induction of ALP expression was detected on day 7. Cells (day 7) were fixed with 4% paraformaldehyde (w/v) at room temperature for 10 min. After washing with PBS and deionized water, cells were stained using BCIP/nitro blue tetrazolium solution for 10 min (56, 57). The results were obtained from three independent experiments. Representative results are shown.

For Alizarin red S staining, WT or TAPT1-deficient C2C12 or C3H/10T1/2 cells were seeded in 24-well culture plates (day 0) and treated with BMP2 protein. The treatment lasted for 21 days and the media with BMP2 were changed every 2 days. The cells were washed with PBS and fixed with 4% paraformaldehyde (w/v) at room temperature for 10 min. After washing with PBS and deionized water, 1 ml of alizarin red S 2% (w/v) (pH 4.1–4.3) solution was added to each well for 30 min. The dye was removed, and the cells were washed with distilled H_2_O (47). The results were obtained from three independent experiments. Representative results are shown.

### Cell fractionation

Cell fractionation was performed as previously described (58). Cell lysates were obtained from transfected or BMP2-treated cells using CTBS buffer (10 mM Tris–HCl at pH 7.4, 140 mM NaCl, 2 mM CaCl_2_) supplemented with 2 mM DTT, 5 mM EDTA, and protease inhibitors. After low-speed centrifugation, the supernatant was used as the primary cytoplasmic separation solution, and the precipitate was used as the primary nucleus. After centrifugation again, the supernatant was used as the finished cytoplasmic separation solution. The precipitate was washed with CTBS buffer and then cleaned by repeated passes through the needle. Finally, the precipitate was lysated with nuclear lysate buffer (CTBS buffer supplemented with 0.2% Triton X-100, 2 mM DTT, 5 mM EDTA, and protease inhibitors). The collected cytoplasmic or nuclear lysates were subjected to immunoblotting or co-IP analysis. The results were obtained from three independent experiments. Representative results are shown in the figures.

### Ectopic ossification

Ectopic bone formation was performed as previously described (59). Briefly, the WT or stable TAPT1-knockdown C3H/10T1/2 cell line was resuspended in PBS. The approximately 5 × 10^6^ cells were injected subcutaneously on both flanks of Athymic nude (nu/nu) mice (4 weeks old, male). After 42 days of feeding, the mice were sacrificed, and the implantations were removed. Implants were subsequently sectioned and stained for H&E staining.

### Statistical analyses

Comparisons between the two groups were performed using Student’s *t*-tests, and data were analyzed with standard errors. Differences among groups were analyzed using GraphPad Prism version 7.01, and significance was defined as *p* < 0.05 or smaller *p* values.

## Data availability

All the data are within the article and supporting information. All the data are to be shared upon request (Jianfeng Zhou or Xiaozhi Rong, Ocean University of China, jfzhou@ouc.edu.cn or rongxiaozhi@ouc.edu.cn).

## Supporting information

This article includes supporting information.

## Conflict of interest

The authors declare that they have no conflicts of interest with the contents of this article.

## Figures and Tables

**Supplementary Figure 2:**